# Elevations of novel cytokines in bacterial meningitis in infants

**DOI:** 10.1371/journal.pone.0181449

**Published:** 2018-02-02

**Authors:** Lakshmi Srinivasan, Laurie Kilpatrick, Samir S. Shah, Soraya Abbasi, Mary C. Harris

**Affiliations:** 1 Department of Pediatrics, The Children’s Hospital of Philadelphia, Philadelphia, PA, United States of America; 2 The Perelman School of Medicine at the University of Pennsylvania; Philadelphia, PA, United States of America; 3 Center for Inflammation, Translational and Clinical Lung Research, Lewis Katz School of Medicine at Temple University, Philadelphia, PA, United States of America; 4 Division of Hospital Medicine, Cincinnati Children’s Hospital Medical Center; Cincinnati, OH, United States of America; 5 University of Cincinnati College of Medicine, Cincinnati, OH, United States of America; 6 Division of Newborn Pediatrics, Pennsylvania Hospital, Philadelphia, PA, United States of America; Universita degli Studi di Parma, ITALY

## Abstract

**Background:**

Bacterial meningitis is challenging to diagnose in infants, especially in the common setting of antibiotic pre-treatment, which diminishes yield of cerebrospinal fluid (CSF) cultures. Prior studies of diagnostic markers have not demonstrated sufficient accuracy. Interleukin-23 (IL-23), interleukin-18 (IL-18) and soluble receptor for advanced glycation end products (sRAGE) possess biologic plausibility, and may be useful as diagnostic markers in bacterial meningitis.

**Methods:**

In a prospective cohort study, we measured IL-23, IL-18 and sRAGE levels in CSF. We compared differences between infected and non-infected infants, and conducted receiver operating characteristic (ROC) analyses to identify individual markers and combinations of markers with the best diagnostic accuracy.

**Results:**

189 infants <6 months, including 8 with bacterial meningitis, 30 without meningitis, and 151 with indeterminate diagnosis (due to antibiotic pretreatment) were included. CSF IL-23, IL-18 and sRAGE levels were significantly elevated in infants with culture proven meningitis. Among individual markers, IL-23 possessed the greatest accuracy for diagnosis of bacterial meningitis (area under the curve (AUC) 0.9698). The combination of all three markers had an AUC of 1.

**Conclusions:**

IL-23, alone and in combination with IL-18 and sRAGE, identified bacterial meningitis with excellent accuracy. Following validation, these markers could aid clinicians in diagnosis of bacterial meningitis and decision-making regarding prolongation of antibiotic therapy.

## Introduction

Neonatal bacterial meningitis is associated with both short and long term adverse consequences, including death and neurodevelopmental impairment [1–3]. The incidence of meningitis is higher in preterm and very low birth weight infants, as well as infants who have experienced invasive instrumentation of their central nervous system, such as myelomeningocele repair or placement of a ventriculoperitoneal shunt [4–6].

There are several challenges to diagnosis of bacterial meningitis in infants. In contrast to adults, signs and symptoms of meningitis are non-specific, and overlap with other non-infectious etiologies [7, 8]. Further, the yield of bacteria from CSF cultures is often low with up to one third of infants demonstrating negative blood cultures [4, 9]. Lumbar punctures (LPs) are often deferred for hours or days after antibiotic administration due to cardiorespiratory instability, or perceived low risk of meningitis, making cultures unreliable [6, 10, 11]. CSF WBC, protein and glucose have only modest sensitivity and specificity for detection of meningitis in this population, highlighting the need for adjunctive diagnostic markers [5, 12]. As a consequence, investigators have evaluated a variety of biomarkers including acute phase reactants and cytokines, and while some have shown promise, none has possessed sufficient diagnostic accuracy to merit widespread clinical use [6, 13, 14].

Most recently several novel cytokines have been identified which play important roles in the initiation and perpetuation of the inflammatory response. IL-23 promotes neutrophil recruitment early in the course of infection, while IL-18 plays a pivotal role in the perpetuation of the inflammatory response [15–17]. The soluble receptor for advanced glycation end products (sRAGE) limits perpetuation of cellular inflammation and damage (including neuroinflammation), prompting its evaluation as a therapeutic target [18–20]. While existing studies show alterations of IL-23, IL-18 and sRAGE in sepsis, with likely biological consequences and potential applicability as diagnostic, prognostic and/or therapeutic targets, these markers have not yet been evaluated in bacterial meningitis [16–18, 21].

These mediators are implicated at different stages of the inflammatory process, and therefore levels of these markers (individually or in combination) may be persistently altered over the first several days of illness, suggesting diagnostic utility. We hypothesized that levels of IL-23, IL-18 and sRAGE are significantly elevated in cerebrospinal fluid of infants with bacterial meningitis as compared to uninfected infants.

## Methods

Study setting: We performed a prospective cohort study of CSF cytokines in bacterial meningitis in three large neonatal intensive care units (NICUs): the Children’s Hospital of Philadelphia, a quarternary center and a regional referral hub, and two inborn level III NICUs, the Hospital of University of Pennsylvania and Pennsylvania Hospital between 2008 and 2012 [6]. Regulatory approval was obtained from the institutional review boards of all 3 participating institutions prior to conduct of the study, and either written or verbal consent was obtained from parents of all study participants.

Study population: Infants < 180 days old receiving a lumbar puncture (LP) or shunt tap for evaluation for meningitis were eligible for inclusion in our study.

Study definitions: Subjects with CSF culture results positive for pathogens and whom clinicians treated with a prolonged course of antibiotics were defined as having *culture proven meningitis*. Subjects whose CSF samples were obtained prior to antibiotic therapy and whose culture results were negative were defined as *negative controls*. Subjects whose CSF culture results were negative, but had received antibiotics prior to the LP, were deemed *indeterminate*, as it was unclear to what extent antibiotic therapy may have affected culture results.

Study procedures: We obtained an additional aliquot of CSF at the time of lumbar puncture or shunt tap performed for clinical reasons. Following informed consent, CSF samples were centrifuged to remove cellular debris, aliquoted to minimize multiple freeze-thaw cycles, and stored in at -80°C.

Data collection: We abstracted demographic, clinical and laboratory data including risk factors for infection, culture results and treatment details from the medical records of enrolled patients. Data were maintained in a coded electronic database.

Sample analysis: Enzyme linked immunosorbent assays were performed for measurement of IL-23, IL-18 and sRAGE levels, using commercially available kits previously validated and utilized in other human studies (Human IL-23 Quantikine ELISA Kit, Human IL-18/IL-1F4 R & D systems, Minneapolis, MN, USA, Human RAGE Quantikine ELISA Kit) [22–24].

Statistical analysis: We computed summary statistics for demographic variables, risk factors, laboratory parameters and therapies. Continuous variables were presented as medians and interquartile ranges (as data were non-parametric), while categorical variables were presented as proportions and/or percentages. We used Wilcoxon rank sum and Kruskal Wallis tests to assess for statistically significant differences between groups. Biomarker levels were correlated with CSF parameters (CSF WBC, protein and glucose) and previously measured CSF cytokine levels (TNF-alpha, IL-1, IL-6, IL-8, IL-10 and IL-12). Receiver Operating Characteristic (ROC) curves were constructed to evaluate the accuracy of individual cytokines in diagnosis of bacterial meningitis. For assessment of accuracy of combinations of cytokines, we employed logistic regression followed by ROC analysis. Finally, we developed cut-off levels based on the best performing individual and combinations markers, which we applied to the indeterminate group, in order to identify subjects who demonstrated elevations of marker levels similar to infants with meningitis. In secondary analyses, we also calculated summary statistics, and performed non-parametric testing and ROC analyses as described above, using a modified dataset. In this modification to the dataset, we utilized the reported lower sensitivity thresholds of the IL-18, IL-23 and RAGE assays to re-classify values below the respective sensitivity thresholds as equivalent to zero.

## Results

A total of 189 subjects were included in this study. Eight subjects had culture proven bacterial meningitis. Thirty infants had negative CSF cultures in the absence of antibiotic pre-treatment and were deemed negative controls; the remaining 151 infants had negative CSF cultures, but received antibiotic pre-treatment and were therefore called indeterminate. Pathogens identified in culture proven meningitis included *Staphylococcus aureus* (n = 4), *Staphylococcus warneri* (n = 1), *Staphylococcus epidermidis* (n = 1), *Enterococcus faecalis* (n = 1) and *Enterobacter cloacae* (n = 1). Infants with culture proven meningitis were more premature, but of greater postnatal age at the time of diagnosis of the infection (Table 1). Fifty-six infants had bloodstream infections at the time of the LP, but the proportion did not differ significantly between subgroups. CSF WBC and CSF protein were significantly elevated and CSF glucose significantly decreased in infants with culture proven meningitis compared with the other groups (Table 1).

**Table 1:** Demographic details and CSF laboratory values.

Variable	Overall cohort (189)	Culture proven meningitis (8)	Negative controls (30)	Indeterminate (151)	P value*
Gestational age (weeks), Median (IQR)	33 (28–39)	30 (24–37)	37 (30–40)	33 (28–39)	0.0683
Birthweight (grams), Median (IQR)	1970 (1060–3160)	1368 (680–2288)	2830 (1531–3176)	1845 (1013–3140)	0.1477
Postnatal age (days), median (IQR)	12 (2–35)	37 (21–50)	16 (2–34)	9 (2–33)	**0.0416**
Preterm^a^, n (%)	119 (63%)	6 (75%)	14 (47%)	99 (66%)	0.114
Neonates^b^, n (%)	132 (70%)	3 (38%)	20 (67%)	109 (72%)	0.105
Male gender, n (%)	122 (65%)	5 (63%)	19 (63%)	98 (65%)	0.979
Race^c^, n (%)	B: 78 (41%); W: 77 (41%); Other: 33 (18%)	B: 1 (12%); W: 5 (63%); Other: 2 (25%)	B: 9 (30%); W: 17 (57%); Other: 4 (13%)	B: 68 (45%); W: 55 (36%); Other: 28 (19%)	0.427
Hispanic ethnicity, n (%)	8 (4%)	0 (0%)	2 (7%)	6 (4%)	0.053
Concurrent culture proven BSI, n (%)	56 (30%)	2 (25%)	4 (13%)	50 (33%)	0.092
Antibiotics prior to LP, n (%)	157 (83%)	7 (88%)	0 (0%)	151 (100%)	**<0.0001**
CSF WBC (cells/mm^3^), median (IQR)	4 (2–11)	104 (29–852)	3 (1–9)	4 (2–9)	**0.0001**
CSF protein (gm/dL), median (IQR)	101 (71–140)	385 (108–783)	96 (59–122)	101 (71–139)	**0.0221**
CSF glucose (mg/dL), median (IQR)	49 (40–58)	26 (20–46)	52 (42–64)	48 (41–57)	**0.0071**

^a^Preterm: Infants born at <37 weeks gestation

^b^Neonates: Infants in the first 28 days of life

^c^B: Black, W: White

*p values based on Kruskal Wallis testing (for continuous variables) and chi squared test (for categorical variables) comparing infants with culture proven meningitis, negative controls and indeterminate subjects

Among the infants with bacterial meningitis, 2 had concurrent bloodstream infections and 1 preterm infant with S. aureus meningitis died. Six infants had prior neurosurgical interventions, (VP shunt placement (n = 4), ventriculostomy (n = 1) and s/p myelomeningocele repair (n = 1)) (S1 and S5 Tables). Of note, the two infants with coagulase-negative staphylococcal isolates were deemed infected by the medical teams caring for them after infectious disease consultation and received a full course of treatment.

**Fig 1:**
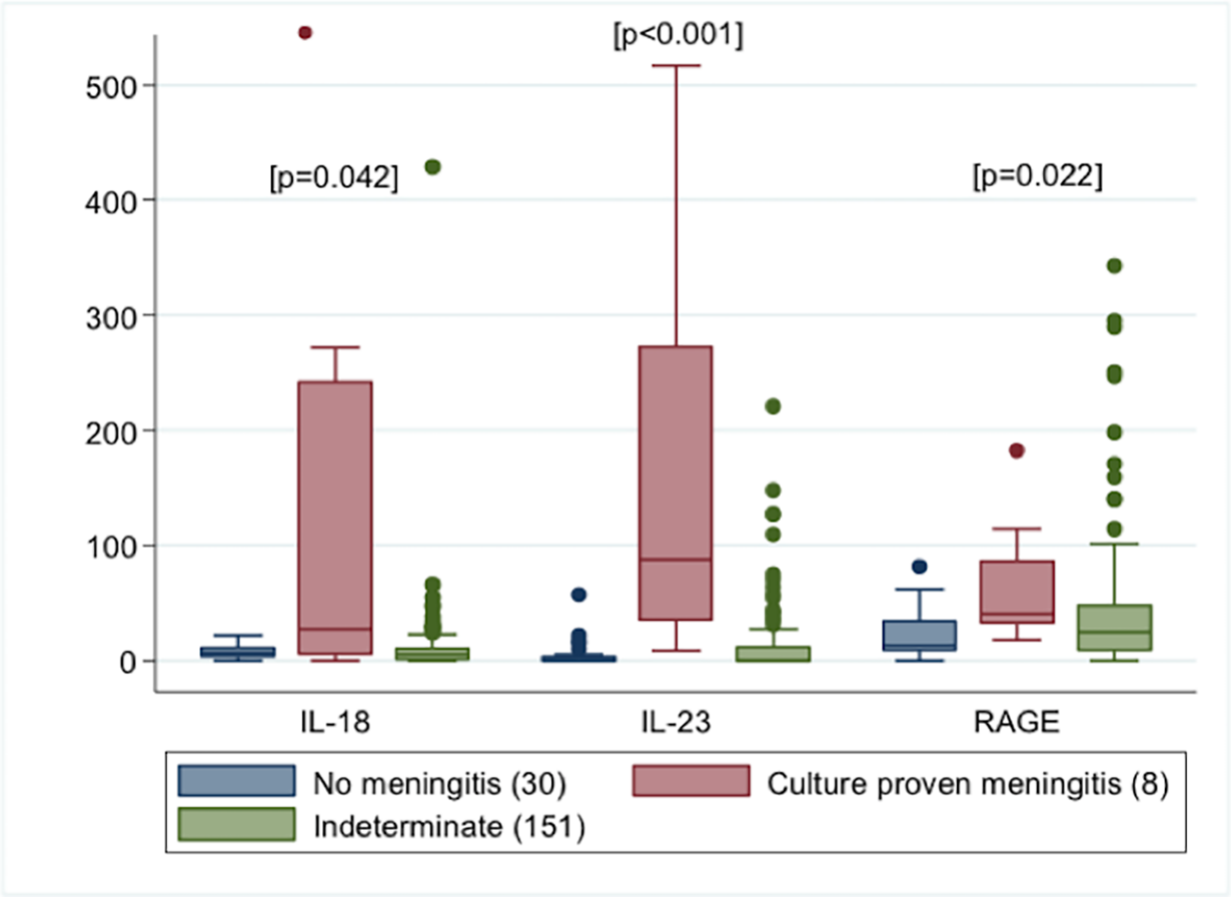
Values of IL-23, RAGE and IL-18 in infants with culture proven meningitis, negative controls and indeterminate subjects. P values based on Kruskal Wallis testing. One outlier value for IL-18 in the infants with culture proven meningitis is represented at the upper bound of the graph as its value greatly exceeded the bounds of the graph (S1 Table provides ranges of values for each marker in each category).

IL-23, IL-18 and sRAGE levels were significantly elevated in infants with culture proven meningitis as compared with the other groups (Fig 1 and S2 Table). All three markers showed significant positive correlation with CSF WBC and protein values, while IL-18 and IL-23 showed negative correlation with CSF glucose levels (Table 2). Though IL-23, IL-18 and sRAGE were significantly correlated with each other, the strength of the correlation coefficients was low. All of these findings remained consistent on secondary analyses using re-classified values based on assay sensitivity thresholds (S6 and S7 Tables).

**Table 2:** Correlation of markers with CSF parameters*.

Marker	CSF WBC	CSF protein	CSF glucose
**IL-18**	0.5307 **(<0.0001)**	0.6308 **(<0.0001)**	-0.2439 **(0.0013)**
**IL-23**	0.2986 **(0.0001)**	0.4393 **(<0.0001)**	-0.2265 **(0.0033)**
**RAGE**	0.1520 **(0.0485)**	0.2062 **(0.0075)**	-0.0140 (0.8573)

*Values represent correlation coefficients of pairwise correlation (significance values in parentheses

**Fig 2:**
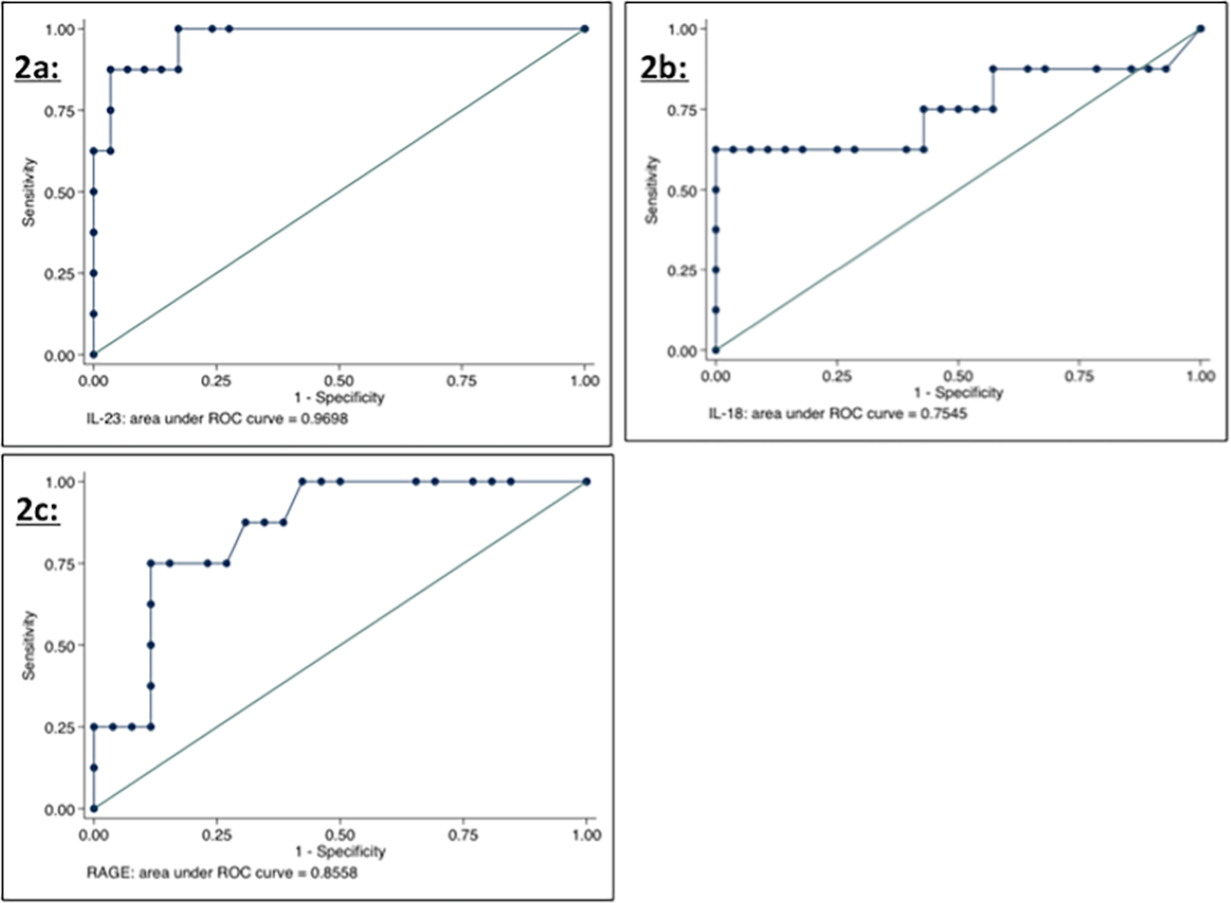
2a: ROC analysis for IL-23; 2b: ROC analysis for IL-18; 2c: ROC analysis for sRAGE.

**Fig 3:**
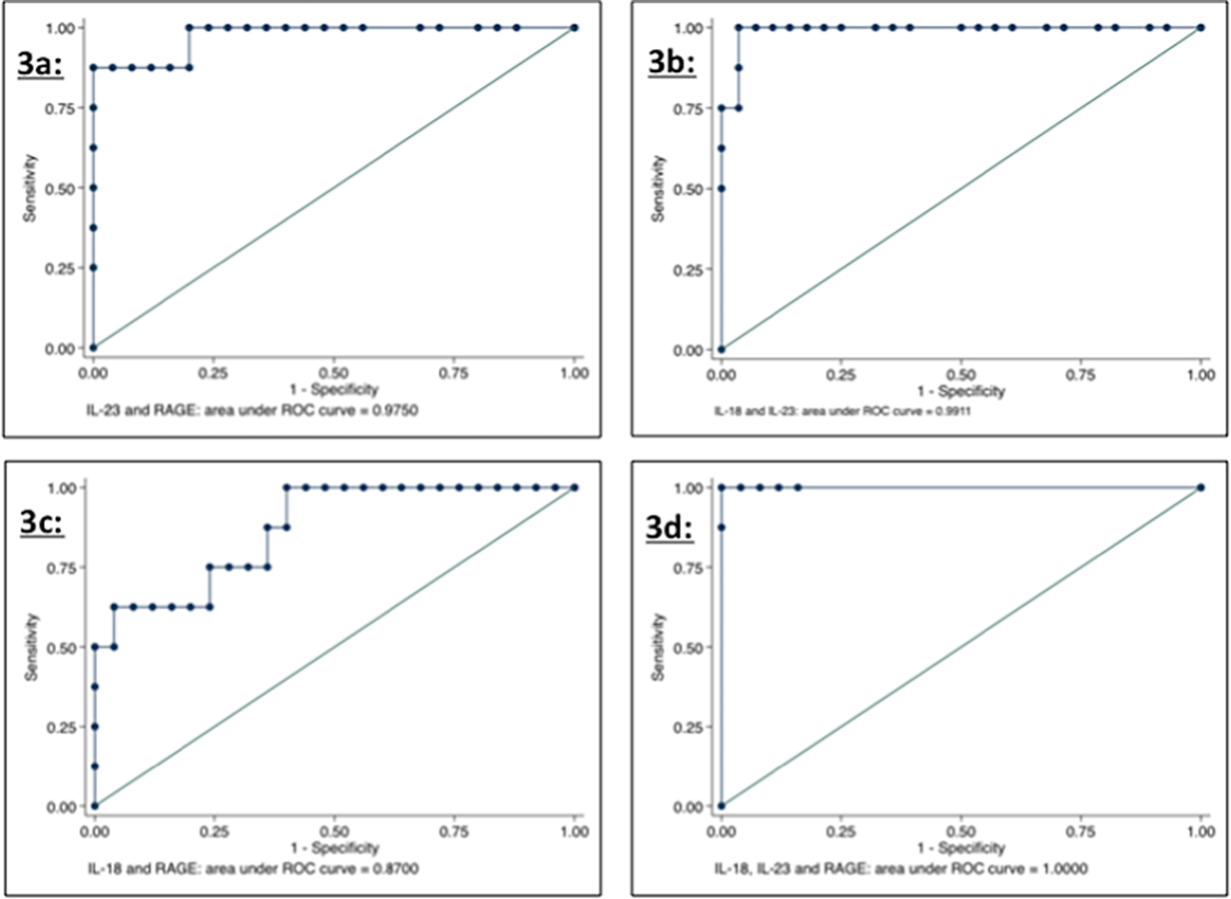
3a: ROC analysis for combination of IL-23 and RAGE; 3b: ROC analysis for combination of IL-23 and IL-18; 3c: ROC analysis for combination of IL-18 and RAGE; 3d: ROC analysis for combination of IL-23, RAGE and IL-18.

When infants with culture proven meningitis were compared to negative controls, IL-23 possessed the greatest accuracy for the diagnosis of bacterial meningitis (AUC 0.9698) (Fig 2A, 2B and 2C); the AUC for IL-23 was better than the results of ROC testing of CSF WBC, protein and glucose (S3 Table), and also better than values obtained for a panel of cytokines in a prior study (S4 Table) [6]. Among combinations of two markers, the use of IL-23 and sRAGE had an AUC of 0.9750, while the use of IL-23 and IL-18 had an AUC of 0.9911 (Fig 3A, 3B and 3C). When all three markers were included as a combined diagnostic strategy, the AUC was 1 (Fig 3D). When these ROC analyses were performed with the modified dataset using re-classified values based on assay sensitivity thresholds, the AUC for IL-23 decreased slightly to 0.9224, but the combinations noted to have high values of AUC on the original analyses continued to demonstrate extremely high accuracy (S8 Table).

In order to better categorize infants with indeterminate CSF culture results due to antibiotic pretreatment prior to CSF sampling, we utilized cut-off levels identified from the ROC analysis. At a cut off value of 8 pg/ml (the lowest level among the culture proven meningitis group) for IL-23, we identified 40 of 151 patients as suspicious for bacterial meningitis. Upon applying a cut-off score generated by weighting all three markers (based on the logistic regression model of the combination of markers), 32 indeterminate individuals were identified as possessing scores similar to infants with culture proven meningitis.

## Discussion

This study provides preliminary evidence of the value of novel biomarkers in the diagnosis of bacterial meningitis in infants. IL-23 demonstrated excellent accuracy in diagnosing meningitis, while the combination of IL-23, IL-18 and sRAGE provides a diagnostic algorithm that identified bacterial meningitis with 100% sensitivity and specificity in this cohort. Following validation, these markers could aid clinicians in decisions surrounding the need for a prolonged course of parenteral antibiotic therapy.

The diagnosis of bacterial meningitis is fraught with uncertainty in infants [9, 25–27]. Limitations in current diagnostic tools include the low yield of CSF culture, which is exacerbated in the setting of antibiotic pre-treatment that occurs frequently in the NICU population[10]. Other parameters such as CSF WBC, protein, or glucose do not possess acceptable sensitivity or specificity with values often overlapping between infected and uninfected infants[5]. This likely causes clinicians to administer prolonged courses of broad-spectrum antibiotics to a proportion of infants with negative CSF cultures, due to concern regarding the adverse consequences of untreated or partially treated meningitis[1, 4, 5, 12]. As such, an ideal adjunctive diagnostic marker should possess optimal sensitivity, to allow clinicians to discontinue antibiotics with confidence, if the test were to return negative, or below the pre-determined cut-off value[10, 28, 29]. We chose to measure IL-23, IL-18 and sRAGE as potential markers with biological plausibility and some evidence suggesting utility in diagnosis of infection.

IL-23 is a pro-inflammatory cytokine with a prominent role early in inflammation [15]. It functions as a mediator of cytokine and chemokine production and recruitment of neutrophils to the site of infection [30, 31]. In prior studies of IL-23 in sepsis, exposure to pathogens was found to lead to increased expression of IL-23 by antigen presenting cells[21, 32, 33]. Our study revealed low levels of IL-23 in uninfected infants; however we identified marked elevations in CSF levels in infants with culture proven meningitis, demonstrating that infants are able to upregulate production of IL-23 in response to infectious and inflammatory stimuli. CSF IL-23 levels correlated with CSF WBC values, and possessed a greater discriminatory ability than CSF WBC in diagnosis of infections, as evidenced by the higher AUC value for IL-23.

IL-18 has also been viewed as a potential therapeutic target in sepsis. IL-18 is most well known for its ability to induce interferon gamma production but also stimulates production of TNF-alpha, IL-1beta, IL-8 and GM-CSF, as well as Th2 cell responses in the appropriate context, thus potentiating and perpetuating the inflammatory response [34–36]. In our study, IL-18 levels were significantly elevated when compared to uninfected infants, although these differences were not as marked as results for the other two cytokines studied. Similarly, the results of its ROC analysis as an individual marker were not as powerful as IL-23. However, the combination of IL-18 with IL-23 and sRAGE showed excellent discrimination for the diagnosis of meningitis.

The receptor for advanced glycation end products (RAGE) functions as a pattern recognition receptor (PRR) that binds to damage associated molecular patterns (DAMPs) and upregulates RAGE expression[37]. sRAGE, the product of proteolytic cleavage of RAGE in response to inflammation, functions as a decoy receptor and binds circulating DAMPs, thus limiting propagation of inflammation [38]. Its levels have been shown to correlate with severity of illness [18, 19, 23]. sRAGE has therefore evoked interest as a potential therapeutic target in SIRS and sepsis [19, 20]. Studies of experimental sepsis and inflammation have shown that RAGE mediated NFkB activation leads to neuro-inflammation, microglial activation, injury to the blood brain barrier and neuronal impairment [39, 40]. In our study, CSF sRAGE levels were found to be elevated in infants with culture proven meningitis compared to controls, thus potentially identifying one mechanism for the adverse neurological consequences of meningitis.

Many of the infants in our study received antibiotics prior to LP, making CSF cultures difficult to interpret. On applying the results of our ROC analysis of both individual and combination markers to the indeterminate group, we were able to further delineate the population who might be at risk of meningitis to about 32–40 subjects. Using this approach, the proportion of infants identified to be at heightened risk in the indeterminate population was similar to the ratio of culture proven meningitis to negative subjects, providing a preliminary measure of face validity to this approach. While these numbers may be an over-estimate of truly infected infants, the use of these combinations of markers could provide utility in defining infants at greatest risk of true meningitic infection and inflammation, and suggest a mechanism for limiting antibiotic exposure in more than two thirds of the indeterminate antibiotic pre-treated subjects.

Our study possesses several strengths. We tested the utility of novel inflammatory markers in the diagnosis of bacterial meningitis, and demonstrated powerful accuracy in ROC analyses, that warrants validation with additional cohorts of subjects. Our study also suggests that the panel of cytokines measured provides excellent diagnostic accuracy when compared with routinely tested CSF parameters, as well as a panel of cytokines previously tested. Secondary analyses applying sensitivity thresholds reported for the assays produced results very similar to the original analysis, demonstrating that these results remain significant and worthy of replication efforts. Furthermore, there is biologic plausibility for the elevations of these markers as they represent successive phases in the initiation and perpetuation of the inflammatory response. Studies of neonatal meningitis are challenging, as this is a rare condition, necessitating enrollment of large cohorts of subjects to achieve a critical number of positive samples. Further, obtaining adequate CSF sample volumes to test multiple markers and replicate findings is often not practicable in this population. Our study faced similar limitations: we acknowledge that our small sample precluded replication of our analyses. Also, our sample size of infants with culture proven meningitis is small, but provides a fair representation of the common pathogens that cause meningitis in tertiary NICUs, especially in populations that have undergone neurosurgical intervention.

In conclusion, we have demonstrated that elevations of IL-23, sRAGE and IL-18 are useful adjunctive markers in diagnosis of bacterial meningitis in infants. These markers could be of especial utility in aiding decision making around discontinuation of antibiotics, thereby potentially decreasing antibiotic use in ‘presumed culture negative meningitis’. These findings require external validation in additional prospective cohorts of infants evaluated for meningitis.

## Supporting information

S1 Table Details of infants with bacterial meningitis. (DOCX)

S2 Table Levels of inflammatory markers in subgroups of infants. (DOCX)

S3 Table ROC areas under the curve for CSF parameters. (DOCX)

S4 Table ROC areas under the curve for previously tested cytokine combinations. (DOCX)

S5 Table Spreadsheet containing raw data used in this study. Includes demographic information, results of clinically obtained laboratory tests and results of ELISA testing for IL-23, IL-18 and RAGE (data also uploaded on Dryad).
(XLSX)

S6 Table Correlation of markers with CSF parameters (using reported sensitivity thresholds of assays). (DOCX)

S7 Table Levels of inflammatory markers in subgroups of infants (using reported sensitivity thresholds of assays). (DOCX)

S8 Table Results of ROC analyses (using reported sensitivity thresholds of assays). (DOCX)
