# Supplementary material for: Elevations of novel cytokines in bacterial meningitis in infants
Source: PLoS One. 2018 Feb 2;13(2):e0181449. doi: 10.1371/journal.pone.0181449 (PMC5796685; doi:10.1371/journal.pone.0181449)
Supplement: S1 Table — (DOCX) [file pone.0181449.s001.docx]

**S1 TABLE: DETAILS OF INFANTS WITH BACTERIAL MENINGITIS**

| **Pathogen in CSF culture** | **PNA***  **(days)** | **GA** (weeks)** | **Birth wt** | **Neurosurgical intervention** | **Bacteremia** | **CSF WBC**  **(/cu.mm)** | **CSF Protein**  **(gm/dL)** | **CSF Glucose**  **(mg/dL)** | **Status at discharge** |
| --- | --- | --- | --- | --- | --- | --- | --- | --- | --- |
| *S. aureus^~^* | 123 | 23.4 | 700 | VP shunt | None | 334 | 75 | 32 | Alive |
| *S. aureus^~^* | 55 | 28 | 1070 | VP shunt | None | 57 | 709 | 20 | Alive |
| *S. aureus^•^* | 40 | 38 | 2540 | Ventriculostomy | None | 29 | 45 | 46 | Alive |
| *S. aureus^•^* | 33 | 24.5 | 600 | S/P MMC repair | None | 1370 | 884 | 20 | Deceased |
| *S. epidermidis* | 44 | 32 | 1665 | VP shunt | None | 1500 | 317 | 19 | Alive |
| *S. warneri*^†^ | 21 | 24.2 | 660 | - | S. warneri | 13 | 141 | 62 | Alive |
| *E. faecalis* | 21 | 35.6 | 2035 | - | E.coli | 30 | 453 | 46 | Alive |
| *E. cloacae* | 15 | 37.5 | 4270 | VP shunt | None | 150 | 856 | 20 | Alive |

*PNA=postnatal age; **GA=gestational age; ^~^MSSA (methicillin sensitive *S. aureus*); ^•^MRSA (methicillin resistant *S. aureus*); ^†^CSF culture positive for S. warneri only in enrichment broth (however based on clinical symptoms and concurrent blood stream infection with same bacteria, decision made to treat for meningitis)
