# Supplementary material for: Elevations of novel cytokines in bacterial meningitis in infants
Source: PLoS One. 2018 Feb 2;13(2):e0181449. doi: 10.1371/journal.pone.0181449 (PMC5796685; doi:10.1371/journal.pone.0181449)
Supplement: S2 Table — (DOCX) [file pone.0181449.s002.docx]

**S2 TABLE: LEVELS OF INFLAMMATORY MARKERS IN SUBGROUPS OF INFANTS**

| **Marker** | **Overall cohort**  **Median (IQR)** | **Culture proven meningitis** | **Negative controls** | **Indeterminate** | **P value*** |
| --- | --- | --- | --- | --- | --- |
| IL-18 | 7 (2.9-12) | 33.1 (7-257.1) | 7.3 (4-11.5) | 6.2 (2.4-11.9) | 0.042 |
| IL-23 | 0 (0-12.3) | 87.6 (35.2-272.5) | 0 (0-3) | 0 (0-11.4) | <0.001 |
| sRAGE | 23 (8-46.1) | 40.6 (32-86.3) | 13 (8-34) | 24.1 (8-48) | 0.022 |

*Kruskal Wallis test
