# Supplementary material for: Elevations of novel cytokines in bacterial meningitis in infants
Source: PLoS One. 2018 Feb 2;13(2):e0181449. doi: 10.1371/journal.pone.0181449 (PMC5796685; doi:10.1371/journal.pone.0181449)
Supplement: S3 Table — (DOCX) [file pone.0181449.s003.docx]

**S3 TABLE: ROC areas under the curve for CSF parameters**

| **Parameter** | **ROC AUC (95% confidence interval)** |
| --- | --- |
| CSF WBC | 0.9312 (0.85336-1) |
| CSF protein | 0.7931 (0.54972-1) |
| CSF glucose | 0.1681 (0-0.34749) |
| CSF WBC + protein | 0.8664 (0.67253-1) |
| CSF WBC + glucose | 0.8319 (0.65264-1) |
| CSF protein + glucose | 0.8534 (0.68946-1) |
| CSF WBC + protein + glucose | 0.8664 (0.70856-1) |
