# Supplementary material for: Elevations of novel cytokines in bacterial meningitis in infants
Source: PLoS One. 2018 Feb 2;13(2):e0181449. doi: 10.1371/journal.pone.0181449 (PMC5796685; doi:10.1371/journal.pone.0181449)
Supplement: S4 Table — (DOCX) [file pone.0181449.s004.docx]

**S4 TABLE: ROC areas under the curve for previously tested cytokine combinations**

| **Cytokine or combination** | **ROC AUC (95% confidence interval)** |
| --- | --- |
| TNF-alpha | 0.8583 (0.69392-1) |
| IL-1 | 0.8208 (0.58984-1) |
| IL-6 | 0.8750 (0.70428-1) |
| IL-8 | 0.8271 (0.65233-1) |
| IL-10 | 0.8854 (0.75162-1) |
| IL-12 | 0.6646 (0.43881-0.89035) |
