# Supplementary material for: Elevations of novel cytokines in bacterial meningitis in infants
Source: PLoS One. 2018 Feb 2;13(2):e0181449. doi: 10.1371/journal.pone.0181449 (PMC5796685; doi:10.1371/journal.pone.0181449)
Supplement: S6 Table — (DOCX) [file pone.0181449.s006.docx]

**S6 Table: Correlation of markers with CSF parameters (using reported sensitivity thresholds of assays)***

| **Marker** | **CSF WBC**** | **CSF protein**** | **CSF glucose**** |
| --- | --- | --- | --- |

| **IL-18**** | 0.5323  (<0.0001) | 0.6346  (<0.0001) | -0.2440  (0.0013) |
| --- | --- | --- | --- |
| **IL-23**** | 0.2997  (0.0001) | 0.4369  (<0.0001) | -0.2272 (0.0031) |
| **RAGE** | 0.1534  (0.0465) | 0.2112  (0.0061) | -0.0219 (0.7786) |

*Values below reported sensitivity thresholds (12.5 pg/ml for IL-18, 16.3 pg/ml for IL-23, 16.14 pg/ml for RAGE) assigned as 0 for the purpose of this analysis

**Values represent correlation coefficients of pairwise correlation (significance values in parentheses)
