# Supplementary material for: Elevations of novel cytokines in bacterial meningitis in infants
Source: PLoS One. 2018 Feb 2;13(2):e0181449. doi: 10.1371/journal.pone.0181449 (PMC5796685; doi:10.1371/journal.pone.0181449)
Supplement: S7 Table — (DOCX) [file pone.0181449.s007.docx]

**S7 Table: Levels of inflammatory markers in subgroups of infants (using reported sensitivity thresholds of assays)***

| **Marker** | **Overall cohort**  **Median (IQR)** | **Culture proven meningitis** | **Negative controls** | **Indeterminate** | **P value**** |
| --- | --- | --- | --- | --- | --- |
| **IL-18** | 0 (0-0) | 33.1 (0-257.1) | 0 (0-0) | 0 (0-0) | 0.003 |
| **IL-23** | 0 (0-0) | 87.6 (35.2-272.5) | 0 (0-0) | 0 (0-0) | 0.001 |
| **sRAGE** | 23 (0-46.1) | 40.6 (32-86.3) | 0 (0-34) | 24.1 (0-48) | 0.007 |

*Values below reported sensitivity thresholds (12.5 pg/ml for IL-18, 16.3 pg/ml for IL-23, 16.14 pg/ml for RAGE) assigned as 0 for the purpose of this analysis **Kruskal Wallis test
