# Supplementary material for: Elevations of novel cytokines in bacterial meningitis in infants
Source: PLoS One. 2018 Feb 2;13(2):e0181449. doi: 10.1371/journal.pone.0181449 (PMC5796685; doi:10.1371/journal.pone.0181449)
Supplement: S8 Table — (DOCX) [file pone.0181449.s008.docx]

**S8 Table: Results of ROC analyses (using reported sensitivity thresholds of assays)*:**

| **Marker (or combination of markers)** | **AUC** |
| --- | --- |
| IL-18 | 0.7790 |
| IL-23 | 0.9224 |
| RAGE | 0.8558 |
| IL-18 + IL-23 | 0.9866 |
| IL-18 + RAGE | 0.8900 |
| IL-23 + RAGE | 0.9800 |
| IL-18 + IL-23 + RAGE | 1.0000 |

*Values below reported sensitivity thresholds (12.5 pg/ml for IL-18, 16.3 pg/ml for IL-23, 16.14 pg/ml for RAGE) assigned as 0 for the purpose of this analysis
